# Ethical Obligation Towards Research Subjects

**DOI:** 10.4103/0973-1229.32153

**Published:** 2007

Chapter V on Foundations and Task Forces discussed issues related to research obligations towards human research subjects. This chapter takes the discussion further and is concerned with the ethical obligations of investigators. The remedy suggested is looking into the contract process for clinical research and following Best Practices Guidelines and Good Publication Practice Guidelines.

## Research Subjects: Our Obligations

An issue of major concern is protection of the interests of research subjects. The reason why patients agree to become research subjects is not only for personal medical benefit but, as an extension, to benefit the rest of the patient population and also advance medical research. These areas are hardly served if research data is doctored or concealed, as can happen to protect industry interests or if industry solely decides the terms and conditions of research contracts.

The ethical obligations of investigators to protect the rights and interests of research participants have been articulated repeatedly (National Commission for the Protection of Human Subjects of Biomedical and Behavioral Research, 1979; Declaration of Helsinki, 1964, updated 2004). Moreover, although informing subjects about risks and benefits empowers them to protect their interests and their rights as decision makers, it is equally the case that patients expect and trust guidance given by their doctors on whether or not to participate in research, which therefore puts added responsibility on researchers to ensure protection of the rights and interests of their research subjects:

It is widely assumed that informing prospective subjects about the risks and possible benefits of research not only protects their rights as autonomous decision makers, but also empowers them to protect their own interests. Yet interviews with patient-subjects conducted under the auspices of the Advisory Committee on Human Radiation Experiments suggest this is not always the case. Patient-subjects often trust their physician to guide them through decisions on research participation. Clinicians, investigators and IRBs must assure that such trust is not misplaced (Kass et al., 1996).

The case of trust is often much more so in countries like India where patient trust may land them in various exploitative situations. Trust is the fragile foundation of contemporary research (Kass *et al*, 1996). Since it is fragile, it needs careful handling. Scientific research is especially prone to manipulation as the temptation to utilise one's power over trusting subjects has a great opportunity to get misused. Science, we know, offers great powers, without the necessary obligation to utilise it responsibly. This is its greatest drawback. Scientific research involving human subjects can be the most tragic example of this truism. Processes inbuilt into research protocols that protect against exploitation need faithful implementation.

## Why Do Patients Participate In Research?

Patients take part in clinical research for a variety of reasons and although many participate with the hope of personal medical benefit, they tend to endorse the notion that they are contributing to scientific knowledge and helping others through eventual improvements in medical care (Kass *et al.*, 1996; Subject interview study, 1996; Sugarman *et al.,* 2001). Indeed, part of the ethical justification for exposing patients to the risks of research that may not offer a personal benefit hinges on the benefit of gaining generalizable knowledge and the assumption that participants understand this (Schulman *et al.,* 2002). If institutions and sponsors fail to ensure publication of the knowledge obtained from the research, they arguably fail to honour their implicit commitment to participants. The results of the study quoted in the previous chapter (p104) suggest that many academic institutions do not guarantee that this commitment will be fulfilled (*ibid).* Which places upon us that much more an obligation to set in processes and safeguards that it does.

An interesting suggestion given by the same authors is how to communicate research information to a wider audience and an important suggestion is about failed trials:

Research findings can be disseminated in various ways, including publication in peer-reviewed journals and posting on the Internet or in public electronic archives. The latter may be an important outlet for trials that failed because of inadequate enrollment or other factors unrelated to efficacy or toxicity (Schulman et al., 2002; International Committee of Medical Journal Editors, 2002).

## The Remedy

### Look Into The Contract Process

The conclusions of Schulman *et al.* (2002) study, as noted earlier, are very clear indeed:

1. Academic institutions routinely engage in research that fails to adhere to ICMJE guidelines for trial design, access to data and publication rights.
2. Our findings suggest that a reevaluation of the process of contracting for clinical research is urgently needed.

The first statement describes a state of affairs, the second a possible solution. The remedy suggested is looking into the contract process for clinical research, obviously ensuring that academia or researchers calls the shots, not industry; or at least researchers can see to it that patient welfare is not compromised even as the profits of industry are to be ensured.

Why can such a via media not be worked out? It can, if industry is ready to give in somewhat and academia ready to assert itself somewhat. In the long run, the interests of both would be served, for genuine research which benefits patients must earn profits for industry and credibility for research. Why can patient welfare not become paramount even from purely selfish business or professional interests? Some elaboration of this is in an editorial elsewhere in this monograph (p11-14; Singh and Singh, 2007) and can be resolved only if we decide where is medicine heading (Singh and Singh, 2005-2006).

### Establish Best Practices Guidelines

Another interesting development needs a brief mention here. The process of setting up ‘best practices’ guidelines for interactions between the pharmaceutical industry and clinicians has already begun and can have important consequences for patient care. Similarly, Good Publication Practice (GPP) for pharmaceutical companies have also been set up aimed at improving the behaviour of drug companies while reporting drug trials (Wager *et al,* 2003).

Some researchers (for example, Steiner *et al*, 2003) have initiated a quality-assurance process in their organization by developing ethical guidelines for such interactions. Guidelines can serve as an important early step toward the ultimate achievement of best practices. Over time, with monitoring of the impact of their guidelines on their institution, they expect academics would understand more about this vital theme. Attempts by other clinical organizations to develop guidelines would be needed to add to a generalized strategy for interactions between the pharmaceutical industry and the clinician.

What Steiner *et al* (2003) found and got concerned about was what many from academia observe quite commonly, but probably do nothing about:

At the Connecticut Mental Health Center, an academic community mental health center jointly run by the State of Connecticut and Yale University, the medical and professional staff became concerned about the increasing presence of pharmaceutical representatives in a variety of activities. There appeared to be an increase in the number of lunches and programs sponsored and catered by the companies as well as an increase in the amount of direct marketing material visible in patient care areas. The level of concern was raised considerably when a company offered to pay a large sum of money for the staff of one program to engage in a day-long “retreat” that had dubious educational goals (Steiner et al, 2003).

What they did was interesting:

Senior medical staff decided to develop a mechanism for tracking all such requests for support and to develop a centerwide policy for future activities. The process, which evolved over the course of one year, produced a flexible set of guidelines that can be applied to a wide range of proposals. The benefit of such an approach is that staff have been engaged in a series of educational discussions about the ethics and merits of pharmaceutical company support for specific projects. We have taken an approach whereby an interdisciplinary committee assesses the risks and benefits of each proposal and makes a final determination of approval or refusal (Steiner et al, 2003).

Based on their experience they developed guidelines for reviewing proposals for pharmaceutical support for clinical and educational activities that evolved as a set of questions rather than a rigid policy. In a manner similar to weighing the risks and benefits of a particular medication or therapeutic intervention, each proposal for support could be viewed as having potential value, which may or may not outweigh any potential drawbacks inherent in the involvement of funding from a for-profit company. A four-person committee was able to reach consensus on 21 proposals by assessing the apparent balance between these factors in their setting (Steiner et al, 2003). They laid down for themselves a set of questions to be asked about each activity, from which, according to them, principles or guidelines easily followed. They were (ibid):

- Will the activity provide direct benefit to patients?
- What incentive does the company have to sponsor the activity?
- Are alternative funding mechanisms available to support the activity?
- If a company is presenting potentially biased information-for example, comparison data that are favorable to its own product-is there an opportunity for other views and data to be presented?
- Is the opportunity for support equitably distributed among staff or programs within the facility?

In other words, they had a best-practices model in place, developed by a medical and professional staff, to navigate them through the complex issues of accepting resources from pharmaceutical companies within the culture of an academic mental health center. By taking a risk-benefit approach to the specific requests and opportunities and by asking relevant ethical and practical questions, their group was able to reach consensus decisions and provide an educational structure from which flexible guidelines were derived. They believe this model provides a conceptual framework for approaching ethical decisions readily adaptable to other settings. Of course they did not hesitate to point out that the model would need to be evaluated over time and in other settings to assess any further positive or negative impact on patient care, the educational programme and inter-professional relationships (Steiner *et al*, 2003).

This is one programme worth a close look and possible replication as to utility in other centers.

## Concluding Remarks

The progress of biomedical research depends on ready availability of research subjects. But such ready availability, in turn, depends on ethical practices by researchers and sponsoring agencies. The clear-cut power to protect research subjects should be inbuilt in the contract process. Establishment of Best Practice Guidelines for researchers and academic medical centers and Good Publication Practice for sponsoring pharmaceuticals, are two important developments worth a close study and replication to assess feasibility across diverse geographical areas.
